# Institutional Notes and News

**Published:** 1910-04-30

**Authors:** 


### Institutional Notes and News.

**Ilford Emergency Hospital.**

For some years now carnivals and other efforts have been made on behalf of this hospital, and a very notable step was taken on April 15, when the Lord Mayor and Lady Mayoress, with the sheriffs, went in state to Ilford to lay the foundation-stone of an institution which should be of the very greatest service in this rapidly growing district. The civic party first went to the Town Hall, where an address was presented by the Ilford U.D.C. Then an adjournment was made to the site of the hospital, which adjoins Newbury Park railway station. Here his Lordship laid the foundation-stone with all due ceremony. Although the weather was not of the best, the event passed off with the greatest success. A silver trowel was presented to the Lord Mayor by Mr. R. Banks-Martin, the architect, and the builder, Mr. H. J. Carter, superintended the lowering of the stone. Amongst those who were prominent in the proceedings were Councillor H. M. Thornton, J.P. (Chairman of the Council), Mr. Benn Bailey, J.P. (Chairman of the Governors of the Hospital), Mr. J. W. Godfrey (Vice-Chairman), Mr. T. Hughes (Treasurer), and Mr. B. Henderson (Hon. Secretary), together with the Town Clerk, Mr. Amos Partington. The next carnival (always one of the best of its kind) on behalf of the hospital will take place on July 9.

**Salop Infirmary.**

A special meeting of the Governors of the Salop Infirmary was held in the board-room on Saturday, April 9, to elect an hon. physician in the place of Dr. Creeton, who, after twenty-nine years' service on the honorary staff of the institution, has resigned. The two candidates were Dr. C. V. Bulstrode and Dr. Frank Whincup. Very keen interest was taken in the appointment, and there was a crowded meeting. On a show of hands the Chairman (Mr. A. Wynne-Corrie, Treasurer of the Infirmary) said he did not feel justified in relying on his power to count the hands, the numbers being so equal. A poll was then demanded, the result of which gave Dr. Bulstrode 116 votes and Dr. Whincup 112. There was a further recount and scrutiny of the papers, and it was then declared that Dr. Bulstrode was appointed, having 113 votes, and Dr. Whincup 107. Subsequently the chairman asked Dr. Creeton to accept the post of consulting physician to the Salop Infirmary, and thanked him for the many years of devoted service which he had given to the institution.

**Woking Cottage Hospital.**

To begin a year with a credit balance of £15 12s. 2d., and to end it with a sum of £145 on deposit and £8 9s. 5d. in hand, is a record of which any hospital may be proud. Yet this is the achievement recorded in the annual statement of the Woking Cottage Hospital. Moreover a considerable sum was spent on improvements and repairs; for instance, outside painting and the gravelling of the drive and garden paths. The twelve beds which the hospital's two general wards contain provided for 243 patients, and the out-patient department was responsible for 643 persons, 105 of whom were dental cases. The medical staff comprises four medical men, and fourteen others from the district have exercised their right, which the hospital's rules allow them, of attending patients who have been under their care previously. Probably the most successful effort during the past year towards raising money was the ball for which Dr. and Mrs. Colby, with Mr. and Mrs. de Courcy Hamilton, and a ladies committee, were responsible. This realised no less than £110.

**Leicester Infirmary.**

At a recent meeting of the Board of Governors of Leicester Infirmary, Sir Edward Wood presiding, the House Committee submitted the following figures of work done for the quarter ending March 31, 1910, as compared with the corresponding period of 1909. There are 59 more in-patients, 558 fewer out-patients, 15 more $x$-ray cases, and 108 more operations than there were last year. The Finance Committee reported that during the same periods the income had shown on the general account, legacies included, a decrease of £505: ordinary income, £4,143 against £3,655; extraordinary income (legacies) £367, against £1,360; but on the children's hospital account there is a healthy increase of £208. The expenditure has been £4,686 6s. 1d., against £5,133 10s. 9d.; and the average daily number of beds occupied 219.5, as compared with 209.

**Gravesend Hospital.**

The hon. treasurer of the Gravesend Hospital, Mr. C. E. Hatten, remarked that the recent report was one of the best they had ever drawn up, but he was compelled to add that this praise referred to the manner of its presentation, and not, alas! to the financial circumstances which it showed. A sum between £400 and £500 will have to be raised every year before income and expenditure balance each other. Princess Alexander of Teck is to visit the hospital this year, and it is hoped that her visit will do what recent fêtes have failed to do—that is, to stimulate local givers to additional and badly wanted generosity. During 1909, 622 in-patients were received, nineteen more than in the previous year, and a record in the hospital's history. In the out-patient department 7,454 persons were treated, which is again a record number, their 30,000 attendances being 5,025 more than in the foregoing year. We are glad to record that a spontaneous and flattering compliment was paid by the auditors, and warmly seconded by Mr. Hatten, to Mr. Pearson for the admirable way in which he keeps the books.

**General Hospital, Herefordshire.**

There is an interesting organisation in connection with the Herefordshire General Hospital, known as the Cresswell Fund, which last year raised something like £500 from the working classes. This fund, which was started by Mr. Cresswell, has a carefully designed machinery and, by the pennies it sets itself out to collect, has formed, and, Sir H. Archer Croft said recently in public, will probably form to a growing degree, one of the staple supports of the hospital. It is the penny subscribers, Sir H. Archer Croft insisted, rather than the disappearing class of old-fashioned writers of cheques, on whom the hospital must lean to-day. He made good point by adding that collectors should not emphasise the individual benefit that penny subscribers might hope for. "I am sure they are very thankful," he added, "if they never want to use the hospital. They do not give with that object: they give to help the county institution." The number of in-patients admitted to the wards was 823 last year, the number of out-patients 4,073, and the daily average number of occupied beds 69. The Bull Convalescent Fund sent nine patients to sanatoria at Weston, Bath, Rhyl, and elsewhere. Without going into financial details we may add that though the ordinary income last year was larger than before, £280 was owing to the bank at the end of it.

